# Supplementary material for: Content-rich biological network constructed by mining PubMed abstracts
Source: BMC Bioinformatics. 2004 Oct 8;5:147. doi: 10.1186/1471-2105-5-147 (PMC528731; doi:10.1186/1471-2105-5-147)
Supplement: Additional File 5 — The original Chilibot query results of the term "long-term potentiation (LTP)" and 22 other terms, limiting the latest references analyzed to the years 1990, 1995, 2000, and 2004. [file 1471-2105-5-147-S5.bz2 › chilibotAdditionalFile5/ltp2000/html/ERK_ZIF268.html]

 


 **ERK** and **ZIF268** 
  
Found 4 abstracts in PubMed,  **4 abstracts were retrieved and analyzed**.  


---

 Search Google  |
 PDF files only 
|  EDU domain only 

---

**Interactive relationship** (e.g. stimulation, inhibition, etc)

**Parallel relationship** (e.g. studied together, co-existance, homology, etc.)

- In this study we examined the contribution of MAPK1 and 2 also known as extracellular signal regulated kinases  **ERK**  1 and 2 to the induction of  **zif268**  mRNA in PC12D cells by using two methods to block the activation of these kinases.  Ref: 10050043 J Biochem (Tokyo), 1999
- Following a similar time course, the two downstream transcriptional targets of MAPK  **ERK** , cAMP response element binding protein CREB and the ternary complex factor Elk 1, a key transcriptional regulator of serum response element SRE driven gene expression, were hyperphosphorylated and the immediate early gene  **zif268**  was upregulated.  Ref: 10844026 J Neurosci, 2000
